# Natural American Spirit brand preference among smokers with mental illness

**DOI:** 10.18332/tid/94456

**Published:** 2018-09-17

**Authors:** Anna E. Epperson, Nicole Anzai, Judith J. Prochaska

**Affiliations:** 1 Stanford Prevention Research Center, Stanford University, Stanford, CA, United States

**Keywords:** mental health disorders, marketing, smoking/ harm reduction, special populations, tobacco industry

## Abstract

**INTRODUCTION:**

Despite a steady decline in the US smoking prevalence over the past 50 years, Natural American Spirit cigarettes (NAS), marketed as ‘natural’ and ‘organic’, have seen a 400% rise in sales. In a sample of smokers with mental illness, based on previous research, we hypothesized that preference for NAS would be associated with younger age, higher education, and a stronger health-orientation.

**METHODS:**

Adult smokers were interviewed during acute psychiatric hospitalization in California between 2009–2013, reporting their preferred top three brands of cigarettes, smoking behaviors, self-rated health, and dietary and physical activity behaviors. The sample (N=956; Mean age=38.7 years, SD=13.5; 48.7% women) identified as 14.5% Hispanic ethnicity, 49.6% White, 23.7% African American, and 23.8% other.

**RESULTS:**

NAS was identified as a top preferred brand by 15.2% of the participants and was the fourth most popular brand for the sample overall. In a multivariate logistic regression, preference for NAS was significantly greater among participants who were younger (OR=0.97), had some college education or more (OR=2.64 to 4.31), ate a low-fat diet (OR=1.56) and reported better overall health (OR=1.26), with p<0.05. Identifying as Hispanic (OR=1.80) or White (OR=3.00) also predicted NAS preference, p<0.05. NAS preference did not differ by gender or psychiatric diagnosis.

**CONCLUSIONS:**

Study findings indicate greater NAS brand appeal among smokers living with mental illness who are younger, more highly educated, and have a stronger orientation to health, perhaps because they perceive NAS to be a ‘healthier’ cigarette to smoke. Marketing language that obscures the harms of smoking ought to be prohibited.

## INTRODUCTION

The prevalence of cigarette smoking among US adults has declined, however, public health gains have not been experienced equally^1^. In particular, the smoking prevalence is two- to four-fold greater among individuals with mental illness relative to the general population, and over 40% of cigarettes sold in the US are sold to persons with mental illness or substance-use disorders^2^. Smokers with mental illness face significant tobacco-related social, economic, and health disparities and are at greater risk of developing heart disease, lung disease, and tobacco-related cancers^2,3^.

Though just as harmful as other commercially available cigarette brands, Natural American Spirit^TM^ (NAS) cigarettes, marketed as ‘natural’ and ‘organic’, have uniquely seen a 400% rise in US sales since 2002^4^. This is a super-premium brand, manufactured by Reynolds American that has a higher average price, but nearly 1 million adult smokers in the US

prefer this brand of cigarettes^5^; the brand is at the top three for sales in California^6^ and has a 10.2% market share in San Francisco^7^.

Previous research indicates that cigarettes marketed as ‘natural’ are perceived as less likely to cause disease and less harmful to overall health^8^. In the national Population Assessment of Tobacco and Health (PATH) Survey, 2.3% of adult smokers reported NAS as their preferred brand, and NAS smokers were 22 times more likely to rate their brand of cigarettes as less harmful relative to smokers of other brands; preference for NAS was more likely among men and younger, White, more highly educated smokers^5^.

Smokers with mental illness represent a large proportion of the tobacco market and have been historically targeted by the tobacco industry^9^. No study to date, however, has examined the extent to which NAS is a brand of preference among smokers with mental illness. Based on the literature in the general population, we hypothesized that NAS would be more popular among younger and better educated smokers with mental illness. Adding further to the literature, we hypothesized that variables that reflect a stronger health orientation would be associated with NAS brand preference in this sample. Specifically, we hypothesized that smokers with mental illness who viewed themselves as healthier or engaging in healthy behaviors (e.g. eating a low-fat diet, exercising) would be more likely to identify NAS as a top brand.

## METHODS

### Participants

Participants were adult smokers (N=956; Mean age=38.7 years, SD=13.5) recruited during psychiatric hospitalization at eight smoke-free acute care units in California as part of a longitudinal clinical trial examining stage-tailored smoking cessation interventions^10^. Eligibility criteria included smoking at least 5 cigarettes per day, prior to hospitalization, and English literacy. All participants provided informed consent, and each participating site’s Institutional Review Board approved the study procedures.

### Measures

Primary psychiatric diagnosis was assessed using the Mini-International Neuropsychiatric Interview Screener, categorized as psychotic disorders, bipolar disorder, unipolar depression, or other^11^. On a single item, participants rated their overall health^12^ from poor (1) to excellent (5). Engagement in regular exercise was assessed as ≥ 5 days per week for ≥ 30 minutes per day^13^. Consumption of a low-fat diet was defined as eating foods such as chicken without the skin and fruits and vegetables. Participants listed their preferred cigarette brand(s), up to a maximum of three brands; reported the usual number of cigarettes smoked per day prior to hospitalization; and the time (in minutes) to first cigarette upon wakening. The last two items were scored and summed to create the Heaviness of Smoking Index with scores^14^ ranging from 0 to 6.

### Analysis

Cross-tabulations examined preference for NAS brand cigarettes by sociodemographic characteristics, health behaviors, and smoking characteristics. Multivariate logistic regression models, controlling for sociodemographic covariates, were run to analyze the association between health and smoking-related behaviors with preference for NAS brand cigarettes as one of their top preferred brands.

## RESULTS

NAS was a top preferred brand for 15.2% of the sample, and 10.6% of respondents listed NAS as their only or number one preferred brand. The majority of participants (58.4%) identified two or three preferred brands. NAS was the fourth most popular brand in the sample overall, behind: 1) Marlboro, 2) Newport, and 3) Camel.

In univariate analyses, all sociodemographic covariates, except for gender, were associated with preference for NAS brand cigarettes. Specifically, those who preferred NAS brand cigarettes were more likely to identify as Hispanic or White; had some college education or more; consumed a low-fat diet; and engaged in regular exercise (Table 1). Participants who reported preference for NAS brand cigarettes also were younger (M=34.8 years, SD=13.3 vs M=39.4, SD=13.4) reported being in better health (M=3.1, SD=1.1 vs M=2.8, SD=1.2), and had a lower Heaviness of Smoking Index (M=2.6, SD=1.4 vs M=3.1, SD=1.5), compared to those who preferred other cigarette brands, p<0.05.

**Table 1:** Sample descriptive characteristics (N=956)

	*Overall sample*	*Prefer NAS brand cigarettes*
	*n*	*%*
**Gender**		
Men	482	15.8
Women	466	14.4
Transgender	8	25.0
**Ethnicity**		
Hispanic	139	20.9^a^
Non-Hispanic	777	14.0^b^
**Race**		
African-American/Black	227	4.4^b^
Other	228	18.9^a^
White	475	18.7^a^
**Education status**		
Lower than High school	165	7.3^a^
High school	218	10.1^a^
Some college	372	18.3^b^
College or more	191	22.8^b^
**Primary psychiatric diagnosis**		
Unipolar depression	256	12.1
Bipolar disorder	301	18.3
Psychotic disorder	253	13.8
Other	139	17.3
**Healthy behaviors**		
**Eats a low-fat diet**		
No	537	12.5^a^
Yes	406	19.0^b^
**Engages in regular exercise**		
No	505	12.5^a^
Yes	438	18.6^b^

NAS: Natural American Spirit. a,b Indicate that comparisons are statistically significant at p<0.05, two-tailed test (χ^2^ or independent samples t-test).

In the multivariate logistic regression model, younger age, Hispanic ethnicity, and attending college significantly related to a preference for NAS (Table 2). White participants were significantly more likely to prefer NAS cigarettes than African-American/Black participants. Controlling for these sociodemographic covariates, health-oriented measures of eating a low-fat diet and being in better general health remained significantly associated

**Table 2:** Multivariate logistic regression model for preference of Natural American Spirit brand cigarettes (N = 856)

*Characteristics*	*OR (95% CI)*
**Age**	0.97 (0.96–0.99) *
**Gender**	
Men (reference)	1.00
Women	0.92 (0.61–1.38)
**Ethnicity**	
Non-Hispanic (reference)	1.00
Hispanic	1.80 (1.07–3.02) *
**Race**	
African-American (reference)	1.00
Other	2.33 (1.04–5.19) *
White	3.00 (1.43–6.33) *
**Education status**	
Lower than High school (reference)	1.00
High school	1.35 (0.59–3.07)
Some college	2.64 (1.26–5.53) *
College or more	4.31 (1.92–9.67) *
**Primary psychiatric diagnosis**	
Unipolar depression (reference)	1.00
Bipolar disorder	1.40 (0.83–2.36)
Other	1.18 (0.62–2.24)
Psychotic disorder	1.33 (0.74–2.39)
**Overall self-reported health**	1.26 (1.06–1.51) *
**Healthy behaviors**	
**Eats a low-fat diet**	
No (reference)	1.00
Yes	1.56 (1.03–2.35) *
**Engages in regular exercise**	
No (reference)	1.00
Yes	1.17 (0.78–1.76)
Heaviness of smoking index	0.89 (0.77–1.02)

OR: odds ratio, CI: confidence interval.

* Statistically significant at p<0.05, controlling for covariates in the Table.

with a preference for NAS. Psychiatric diagnosis, engagement in regular exercise, and Heaviness of Smoking were not significant contributors in the multivariate model. The full model’s McFadden’s R^2^ was 0.12, χ^2^(15)=88.49, and p<0.01.

## DISCUSSION

In a large sample of smokers living with mental illness, being of younger age, identifying as Hispanic or White, having some college education, reporting better overall health and eating a low-fat diet were factors significantly associated with preferring NAS cigarettes to other brands. Our findings that preference for NAS is associated with being younger, more highly educated and White are in line with previous findings on preference for NAS in a large national sample^5^. Unlike the prior study, NAS preference did not differ by gender and was greater among participants identifying as Hispanic.

We found that preference for NAS was associated with better health status and engagement in health supporting behaviors. Smokers with a stronger health-orientation may be more likely to prefer the cigarette brand that they perceive as ‘healthier’. This interpretation is supported by findings from several prior studies that have indicated that NAS cigarettes are perceived to be significantly less harmful compared to other brands^5,8^.

Based on unit sales, NAS holds approximately 1.7% of the cigarette market nationally^4^, 5.8% in California^6^, and 10.2% in San Francisco^7^. In the current study, the NAS brand was preferred by 15% of the sample and was the fourth most popular brand overall. The findings suggest that the NAS marketing strategy is having success among smokers with mental illness in Northern California.

In examining brand preference, participants were able to list up to three brands, and most listed more than a single preferred brand. Given the higher price of NAS cigarettes, it may be that some participants prefer NAS, but they also smoke lesser expensive bands, particularly when finances are short.

### Limitations

Generalizability of study findings may be limited, due to the geographical extent. The data were also cross-sectional, thus limiting causal inferences. Participants were not asked about their perceptions of particular cigarette brands, which has been done in prior studies. The current findings contribute to the growing literature on health-oriented marketing of the NAS brand and smoking preferences, with a focus on a vulnerable group.

Consistent with longstanding efforts by the tobacco industry to decrease negative public perceptions of cigarettes and encourage smokers to ‘switch’ (change brand) rather than ‘quit’ smoking^1,15^, NAS cigarettes have been marketed as less risky to health.

## CONCLUSIONS

Study findings indicate greater NAS brand appeal among smokers who are younger, more highly educated, and have a stronger orientation to health. The findings provide further support for comprehensive tobacco regulation that prevents the use of misleading health-oriented terms, such as ‘natural’ and ‘organic’. All commercially available cigarettes in the US are designed to create and sustain addiction and will kill more than half of long-term users if smoked as intended^1^. Marketing language that obscures these health harms among the general population, particularly in vulnerable groups, as seen here, should be prohibited.

## CONFLICTS OF INTEREST

A. E. Epperson reports grants from National Institutes of Health, National Heart, Lung and Blood Institute (#HL007034), outside the submitted work. J. J. Prochaska reports personal fees from pharmaceutical companies that make smoking cessation drugs (Pfizer, Achieve Life Sciences), personal fees from technology companies that make tobacco cessation treatments (Carrot, MD Revolution), personal fees from plaintiffs’ counsel in law suits filed against the tobacco companies, outside the submitted work. N. Anzai has also completed and submitted an ICMJE form for disclosure of potential conflicts of interest. The authors declare that they have no competing interests, financial or otherwise, related to the current work.
